# Suitable CO_2_ Solubility Models for Determination of the CO_2_ Removal Performance of Oxygenators

**DOI:** 10.3390/bioengineering8030033

**Published:** 2021-03-02

**Authors:** Benjamin Lukitsch, Paul Ecker, Martin Elenkov, Christoph Janeczek, Christian Jordan, Claus G. Krenn, Roman Ullrich, Margit Gfoehler, Michael Harasek

**Affiliations:** 1 Institute of Chemical, Environmental and Bioscience Engineering, TU Wien, 1060 Vienna, Austria; paul.ecker@tuwien.ac.at (P.E.); christian.jordan@tuwien.ac.at (C.J.); michael.harasek@tuwien.ac.at (M.H.); 2 CCORE Technology GmbH, 1040 Vienna, Austria; martin.elenkov@tuwien.ac.at (M.E.); christoph.janeczek@tuwien.ac.at (C.J.); claus.krenn@meduniwien.ac.at (C.G.K.); roman.ullrich@meduniwien.ac.at (R.U.); 3 Institute of Engineering Design and Product Development, TU Wien, 1060 Vienna, Austria; margit.gfoehler@tuwien.ac.at; 4 Department of Anaesthesia, Intensive Care Medicine and Pain Medicine, Medical University of Vienna, 1090 Vienna, Austria

**Keywords:** blood oxygenator, carbon dioxide (CO_2_) removal, carbon dioxide (CO_2_) solubility model, model performance, model suitability, evaluation, porcine blood, bovine blood

## Abstract

CO_2_ removal via membrane oxygenators during lung protective ventilation has become a reliable clinical technique. For further optimization of oxygenators, accurate prediction of the CO_2_ removal rate is necessary. It can either be determined by measuring the CO_2_ content in the exhaust gas of the oxygenator (sweep flow-based) or using blood gas analyzer data and a CO_2_ solubility model (blood-based). In this study, we determined the CO_2_ removal rate of a prototype oxygenator utilizing both methods in in vitro trials with bovine and in vivo trials with porcine blood. While the sweep flow-based method is reliably accurate, the blood-based method depends on the accuracy of the solubility model. In this work, we quantified performances of four different solubility models by calculating the deviation of the CO_2_ removal rates determined by both methods. Obtained data suggest that the simplest model (Loeppky) performs better than the more complex ones (May, Siggaard-Anderson, and Zierenberg). The models of May, Siggaard-Anderson, and Zierenberg show a significantly better performance for in vitro bovine blood data than for in vivo porcine blood data. Furthermore, the suitability of the Loeppky model parameters for bovine blood (in vitro) and porcine blood (in vivo) is evaluated.

## 1. Introduction

Membrane oxygenators are medical devices used to support or replace the gas exchange provided by the natural lungs. In modern oxygenators, the gas exchange surface is supplied by hollow fiber membrane packings. While blood is pumped through the shell side of the hollow fiber packing, O_2_ is used to sweep the fiber lumen. CO_2_ and O_2_ are exchanged through the membrane following the partial pressure gradient. Consequently, blood is enriched with O_2_ and purged from CO_2_ [1].

Initially, membrane oxygenators were developed to replace the lungs during cardiopulmonary bypass. In cardiopulmonary bypass, the oxygenator has to take over the total metabolically required O_2_ and CO_2_ transfer of 250 and 200 mL/min, respectively [2].

With continuous development of oxygenators, the membrane performance was improved, and bleeding complications minimized. This allowed the application of oxygenators as partial lung support in the management of acute respiratory distress syndrome (ARDS). Patients suffering from ARDS are often treated with lung protective ventilation (LPV). While LPV allows sufficient O_2_ transfer the CO_2_ removal is limited, evoking serious side effects. To circumvent these side effects, oxygenators are increasingly used to provide sufficient CO_2_ removal [3].

As the CO_2_ concentration of venous blood is high (approximately 500 mL CO_2_/L blood), the total metabolic CO_2_ production can potentially be eliminated by clearing a venous blood flow of 500 mL/min of its CO_2_ content [4]. These lower blood flow rates allow for smaller sized vascular access and a wide range of CO_2_ removal techniques such as arteriovenous, venovenous, total, partial, extracorporeal, and intracorporeal CO_2_ removal. This variety of applications has led to a wide field of research activities. However, for further development of reliable oxygenator-based CO_2_ removal techniques accurate measurement of CO_2_ removal is essential.

Principally, there are two possible methods to evaluate the CO_2_ removal performance of an oxygenator. Either by measuring the CO_2_ amount transferred into the off-gas stream–sweep flow-based method or by determining the CO_2_ amount removed from blood–blood-based method. In both methods, the amount of CO_2_ is calculated by the product of flow rate and the CO_2_ concentration difference between the inlet and outlet of the membrane packing.

In the sweep flow-based method, the sweep gas flow rate is commonly measured using a rotameter, a thermal mass flow meter, or a volumetric piston stroke meter. The CO_2_ concentration of the outgoing sweep gas flow can be measured reliably via on-line non-dispersive infrared spectroscopy (NDIR). CO_2_ concentration of the ingoing sweep gas flow can be assumed zero as medical O_2_ is commonly used as sweep fluid.

In the blood-based method, the blood flow is commonly measured using an ultrasonic flow probe [5]. Compared to the sweep flow-based method, the CO_2_ concentration in blood cannot be measured directly. First, blood samples must be drawn manually, which requires sufficient accessibility to the blood flow. Then, relevant blood parameters can be determined using a blood gas analyzer (BGA). The blood parameters allow for calculating the CO_2_ concentration via a CO_2_ solubility model. Multiple models are available and differ in their complexity and number of inlet parameters. The most common inlet parameters, which can be provided by the BGA, are the CO_2_ partial pressure, pH, hematocrit, and bicarbonate concentration (Section 2.4). In contrast to the sweep flow-based method, the blood-based method requires the CO_2_ concentration to be determined at the outlet and the inlet of the oxygenator. The CO_2_ concentration difference, necessary to calculate the CO_2_ removal, therefore is prone to measurement errors of both inlet and outlet sample values. Furthermore, the CO_2_ solubility models and their additional input parameters, which are not required for the sweep flow-based method, introduce further measurement inaccuracies. Hence, the sweep flow-based method can be considered more stable and accurate (Section 3.1).

Nevertheless, BGA measurements are routinely required to control and correct physiologically or clinically relevant pathological conditions in the blood. This makes a BGA necessary for both sweep flow- and blood-based methods. Consequently, the experimental setup of the blood-based method is less extensive. As a result, both methods were applied in recent research.

The sweep flow-based method is used in several different studies investigating the CO_2_ removal performance of oxygenators due to its relatively simple and accurate measuring principle. Arazawa et al. [6] immobilized carbonic anhydrase on hollow fiber membranes and investigated the impact on the CO_2_ removal performance of the fibers. Experiments were conducted in vitro with phosphate buffered solution (PBS) and bovine blood. The sweep flow-based method allowed a reliable comparison between PBS and bovine blood, which otherwise would have needed different solubility models for the different fluids (PBS and bovine blood). Eash et al. [7] determined the CO_2_ removal performance of a respiratory catheter. The performance was assessed in in vivo trials with sheep and calves as animal model. As the catheters were positioned close to the right atrium, the sweep flow-based method was necessary to overcome a lack of accessibility. Mihelc et al. [8] evaluated different designs for an intravenous membrane catheter by conducting in vitro trials with water and in vivo trials with calves, facing similar challenges as the previously mentioned authors. The sweep flow-based method was also used when no limitations in accessibility or different blood models were present. For instance, May et al. [9] tested the in vitro CO_2_ removal of a low flow membrane oxygenator using bovine blood. Wang et al. [10] tested the gas exchange performance of perfluorocopolymer coated microporous hollow fibers in in vivo trials, using a sheep animal model.

The blood-based method is applied for various reasons. May et al. [11] studied traditional hemodialysis membrane modules for bicarbonate and consequently CO_2_ removal from blood (respiratory hemodialysis). The tests were conducted as in vitro trials with bovine and porcine blood. As the sweep fluid in hemodialysis is liquid dialysate, the conventional NDIR measurement is not applicable. In addition, BGA data at the inlet and outlet provide important blood parameters for the setup of gas exchange simulations. Simulations enable detailed insight into underlying phenomena of the gas exchange and can supplement experimental data which are often limited due to accessibility or regarding spatial resolution. However, a prerequisite for reliable simulations is an accurate solubility model. Hormes et al. [12] developed a micro membrane oxygenator and used BGA measurements and the blood-based method for the setup of a computational fluid dynamics (CFD) model. The blood-based method is also used for examining the CO_2_ removal performance of prototype devices. Schraven et al. [13] evaluated the effects of pulsatile blood flow on CO_2_ removal. For this study in vitro tests with porcine blood were conducted. Wu et al. [14] tested the gas exchange performance of a microfluidic oxygenator with a porous polycarbonate membrane. The CO_2_ removal was determined in vitro with human blood. Borchardt et al. [15] examined an oxygenator with integrated pulsatile pump in in vitro tests using porcine blood.

While both methods have been commonly used in recent research, the results of blood-based and sweep flow-based methods are rarely combined or compared against each other. Barret et al. [16] used both methods to determine the performance of an extra-corporeal CO_2_ removal device. The CO_2_ removal was measured in vitro with human blood, sweep flows variating from 0 to 1000 mL/min, and at a constant blood flow rate (400 mL/min). The relative deviation of blood-based CO_2_ removal from sweep flow-based CO_2_ removal was found to be largest (20%) at low CO_2_ removal rates (57.9 mL/min–lowest sweep flow) and lowest (6%) at high CO_2_ removal rates (94.0 mL/min–highest sweep flow). Average relative deviation was found to be 11%. This indicates that high CO_2_ removal rates are beneficial for the accuracy of the blood-based method. Furthermore, it was favorable that this study was conducted with human blood, the medium most CO_2_ solubility models refer to.

To summarize, oxygenator-based CO_2_ removal is a highly relevant clinical technique. To foster further development, accurate prediction of the CO_2_ removal performance is of great importance. CO_2_ removal can be determined by the accurate sweep flow-based or the less accurate blood-based prediction method. In order to guarantee a reasonable performance of the blood-based prediction method, the selection of an adequate CO_2_ solubility model is crucial. In addition, accurate solubility models are needed for reliable gas exchange simulations. In this research, we compared four different CO_2_ solubility models for blood in a series of experiments conducted to determine the CO_2_ removal of a prototype oxygenator. The experiments comprised in vitro trials with bovine blood and water as well as in vivo trials with pigs as large animal model. The respective CO_2_ removal rates were determined with two different approaches, the sweep flow- and the blood-based method. By comparing the results of both methods, the accuracy of the different solubility models was evaluated. Additionally, a possible adaptation of the empirical Loeppky model parameters to in vitro bovine and in vivo porcine blood was examined. The general performance of the blood-based CO_2_ removal prediction method is discussed.

## 2. Materials and Methods

In vitro trials with bovine blood and water and in vivo trials with pigs as large animal model were conducted to examine the CO_2_ removal of a prototype oxygenator. A parameter study was performed to investigate the influence of CO_2_ partial pressure and blood flow on CO_2_ removal. The CO_2_ partial pressure of the blood entering the prototype oxygenator was adjusted to three different levels (50, 70, 100 mmHg). For each partial pressure three blood flows (1000, 1300, 1600 mL/min) were tested.

The prototype oxygenator incorporated commercial Polymethylpentene (PMP) hollow fibers (Membrana Oxyplus^®^ 90/200 PMP, 3M) and provided a membrane area of 0.06 m^2^. All tests were approved by the institutional ethics and animal welfare committee and the national authority (ZI. 153/115-97/98). The CO_2_-removal performance was determined based on the CO_2_ concentration increase in sweep flow and the CO_2_ concentration decrease in blood.

### 2.1. In Vitro Trials

**Figure 1:**
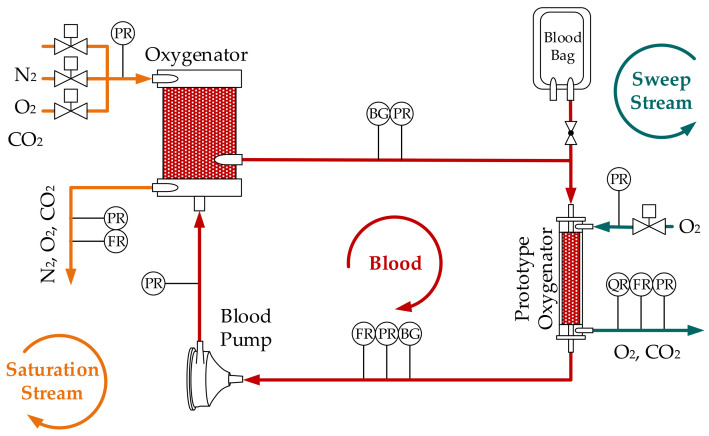
Scheme of the in vitro test setup with prototype oxygenator, blood pump, pressure sensors (PR), flow rate sensors (FR), blood gas analyzer (BGA) sample ports (BG), and CO_2_ concentration sensor (QR).

In vitro experiments were carried out with bovine blood and water. Experiments with water were conducted to gain an estimation for the highest possible accuracy of the blood-based CO_2_ removal prediction (Section 3.2). Bovine blood was provided by the Teaching and Research Farm of the University of Veterinary Medicine, Vienna. The blood was pumped by a rotary blood pump (BPX-80, Medtronic) in a closed circuit from an extracorporeal membrane oxygenator (ECMO Adult, Eurosets) to the prototype oxygenator and back (Figure 1). The blood flow rate was measured with a clamp-on ultrasound flow probe (SONOFLOW CO.55/080). Blood temperature was adjusted to 37 °C using the ECMO heat exchanger. The ECMO fiber lumens were swept with a N_2_/O_2_/CO_2_ saturation stream to enrich the blood with CO_2_ and to adjust pathological venous conditions. The CO_2_ was then removed from the blood in the prototype module. Three blood samples were taken for each measurement point before and after the prototype module. Complying with good clinical practice [17], pre-samples were drawn to clean the line before taking blood samples. The blood samples (4 mL) were drawn in heparinized syringes with constant drag effort. Air bubbles were removed from the syringe and the syringe was closed with an airtight lid. After gentle mixing by repeated inversion, the sample was immediately analyzed with a blood gas analyzer (BGA–ABL825 FLEX, Radiometer Medical A/S). The fiber lumen of the prototype module were swept with pure O_2_ (1 L STP/min) to remove any CO_2_ separated from blood from the test circuit. The sweep gas flow of the ECMO and the prototype oxygenator were controlled by four mass flow controllers (GF40, Brooks). The flow rates of the outgoing sweep gas streams were recorded with volumetric piston stroke meters (Defender 510, Bios DryCal). In addition, the CO_2_ concentration of the sweep gas flow exiting the prototype module was measured via NDIR (BINOS 100 M, Emerson). The absolute pressure was measured before and after the modules on the blood and gas side (PR, see Figure 1), using miniaturized pressure transmitters (AMS 4711, Analog Microelectronics).

### 2.2. In Vivo Trials

**Figure 2:**
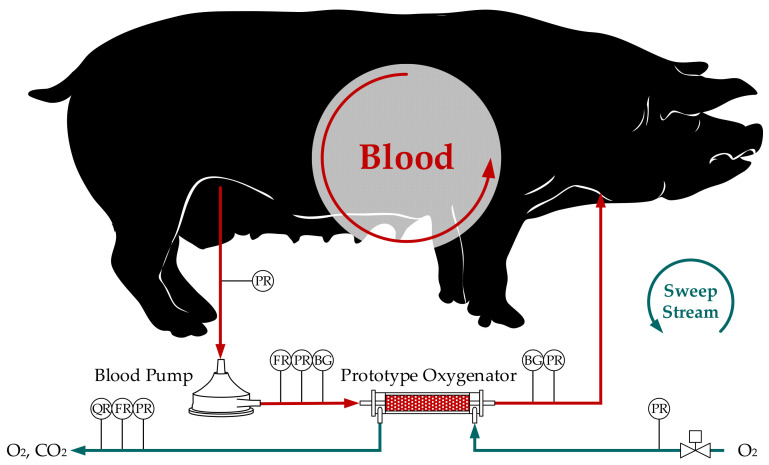
Scheme of the in vivo test setup with prototype oxygenator, blood pump, pressure sensors (PR), flow rate sensors (FR), BGA sample ports (BG) and CO_2_ concentration sensor (QR)–by Lukitsch et al. [18], (CC BY 4.0).

The in vivo study results displayed in this study represent a secondary analysis of previously published data. This preceding publication investigated the prediction of the CO_2_ removal rate of oxygenators using CFD simulations [18]. For validation of the CFD model, the CO_2_ removal predicted by CFD was compared to the CO_2_ removal predicted by the sweep flow-based method. A comparison of sweep flow- and blood-based methods and thus an evaluation of different CO_2_ solubility models was not conducted. The in vivo tests were performed with two pigs provided by the Teaching and Research Farm of the University of Veterinary Medicine, Vienna. Animals were sedated and mechanically ventilated via an endotracheal tube. Arterial oxygenation and CO_2_ partial pressure were controlled by a mechanical ventilator (Servo 900C, Siemens). Ringer’s solution was administered to replace fluid losses and to maintain blood pressure. To prevent blood coagulation, which could lead to clotting in the hollow fiber bundle, heparin was injected intravenously. Blood pressures, cardiac output, body temperature (approx. 37 °C), and heart rate were monitored continuously (PiCCO plus, Pulsion Medical System). Blood was pumped (BPX-80, Medtronic) from the femoral vein (17Fr cannula, Medtronic, USA) into a prototype oxygenator and returned through the jugular vein (19Fr cannula, Medtronic, USA) (Figure 2). The blood flow rate was measured with a clamp-on ultrasound flow probe (SONOFLOW CO.55/080). CO_2_ was removed from the blood flowing on the shell side of the prototype oxygenator. Analogously to the bovine blood experiments, three blood samples were taken for each measurement point before and after the prototype module (Section 2.1). The blood samples were then immediately analyzed with a blood gas analyzer (BGA–ABL825 FLEX, Radiometer Medical A/S). The fiber lumen of the prototype module were swept with pure O_2_ (1 L STP/min) to remove any CO_2_ separated from the blood from the circuit. The sweep gas flow of the prototype oxygenator was controlled by a mass flow controller (GF40, Brooks). The flow rate of the outgoing sweep gas flow was recorded with a volumetric piston stroke meter (Defender 510, Bios DryCal). In addition, the CO_2_ concentration of the sweep gas stream exiting the prototype module was measured via NDIR (BINOS 100 M, Emerson). The absolute pressure was measured before and after the modules on the blood and gas side (PR, Figure 2), using miniaturized pressure transmitters (AMS 4711, Analog Microelectronics).

### 2.3. Determination of CO_2_ Removal

The CO_2_ removal (Q_CO2.i_) is calculated by the product of flow rate (Q_i_) and the CO_2_ concentration difference between the inlet and outlet of the membrane packing (Δc_CO2,i_). This can be done for the sweep fluid (Q_CO2,sweep_) and blood (Q_CO2,blood_):

Q_CO2,i_ = |Q_i_ × Δc_CO2,i_| (1)

In contrast to the CO_2_ concentration in the sweep fluid, the CO_2_ concentration in blood cannot be measured directly and must be computed using BGA measurement data and a CO_2_ solubility model. As the sweep flow-based CO_2_ removal determination was considered as the more reliable and accurate method (Section 3.1), we evaluated the suitability and performances of the different CO_2_ solubility models by using the relative deviation (ε) of the blood-based CO_2_ removal (Q_CO2,blood_) from the sweep flow-based CO_2_ removal (Q_CO2,sweep_).

ε = |Q_CO2.blood_ − Q_CO2.sweep_| ÷ Q_CO2.sweep_ (2)

However, in order to quantify the errors introduced by the solubility model, measurement errors on the blood side (e.g., BGA measurement errors) have to be considered (Section 3.2).

### 2.4. CO_2_ Solubility Models

In this study, four models with different levels of complexity were applied to describe the solubility behavior of CO_2_ in blood. The solubility of CO_2_ in water was modelled using Henry’s law.

#### 2.4.1. Henry’s Law

The Henry coefficient (k_H_), representing the solubility of CO_2_ in water at a temperature (T) of 310.15 K, can be calculated using Equation (3) [19].

k_H_(T) = 0.034 [mol/kg/bar] × exp(2400 [K] × ((1÷T [K]) − (1÷ 298.15 [K]))) (3)

CO_2_ concentration in mL STP CO_2_/mL (c_CO2_) can then be calculated with the partial pressure of CO_2_ (p_CO2_), the density of CO_2_ (ρ_CO2_) at standard temperature and pressure (STP–1 bar, 237.15 K), the molar mass of CO_2_ (M_CO2_), and the density of water (ρ_water_):

c_CO2_ [mL STP CO_2_/mL] = k_H_ (37 °C) × M_CO2_ ÷ ρ_CO2_ × p_CO2_ × ρ_water_ = 0.025 [mol/kg/bar] × 44.01 × 10^−3^ [kg/mol] ÷ 1.784 [kg/m^3^] × p_CO2_ [bar] × 1000 [kg/m^3^] (4)

#### 2.4.2. Loeppky et al.

The simplest model to describe the total CO_2_ content (c_CO2,total_) of human whole blood was proposed by Loeppky et al. [20]. It is given by an exponential equation including CO_2_ partial pressure (p_CO2_) and two regression parameters:

c_CO2,total_ [mL CO_2_/mL] = q × p_CO2_^t^ = 0.128 [mL CO_2_/mL/mmHg^(0.369 [−])^] × (p_CO2_ [mmHg])^0.369 [^^−]^ (5)

#### 2.4.3. May et al.

May et al. [11] calculated the total CO_2_ concentration as a sum of the bicarbonate concentration (c_HCO3_^−^) and physically dissolved CO_2_. For physically dissolved CO_2_, the product of a Henry coefficient for the CO_2_ solubility in blood (K_s_) and CO_2_ partial pressure is used. For the bicarbonate concentration, the value determined by the BGA is used. The BGA, as required by May, determines the bicarbonate concentration based on the Henderson-Hasselbalch equation (Section 3.4) [21].

c_CO2,total_ [mL CO_2_/mL] = (c_HCO3_^−^ + K_s_ × p_CO2_) × V_M_ = (c_HCO3_^−^ [mmol CO_2_/mL] + 3.07 × 10^−^^5^ [mmol CO_2_/mmHg/mL] × p_CO2_ [mmHg]) × 22.56 [mL CO_2_/mmol CO_2_] (6)

The value of K_s_ with 0.023 mol CO_2_/kg/bar is close to the value of the Henry constant for water (0.025 mol CO_2_/kg/bar). V_M_ represents the molar volume of CO_2_ at STP.

#### 2.4.4. Siggaard-Andersen et al.

Siggaard-Andersen et al. [22] also determined the CO_2_ content of whole blood by calculating the sum of dissolved CO_2_ and bicarbonate concentration. This is done separately for blood plasma and red blood cells. The CO_2_ concentration of blood plasma (pl) can be computed using Equation (7). Dissolved CO_2_ is calculated based on a solubility coefficient (α_CO2,pl_) and p_CO2_. The determination of bicarbonate concentration additionally incorporates the pH of plasma (pH_pl_) and the negative logarithmic equilibrium constant for the overall CO_2_ hydration reaction in blood plasma (pK_pl_):

c_CO2,pl_ [mmol CO_2_/L] = α_CO2,pl_ × p_CO2_ × (1 + antilg(pH_pl_ − pK_pl_)) = 0.230 [mmol CO_2_/L/kPa] × p_CO2_ [kPa] × (1 + antilg(pH_pl_ [−] − pK_pl_ [−])) (7)

Solubility of CO_2_ in plasma (α_pl,CO2_) with a value of approximately 3.07 × 10^−5^ mol/mmHg/L is comparable to the parameter K_s_ (May model, Equation (6)). The negative logarithmic equilibrium constant in blood plasma (pK_pl_) can either be assumed constant (6.10) or determined by using Equation (8). This study uses the calculation method of Equation (8) proposed by Siggaard-Anderson et al.

pK_pl_ = 6.125 − lg(1 + antilg(pH_pl_ − 8.7)) = 6.125 [−] − lg(1 + antilg(pH_pl_ [−] − 8.7 [−])) (8)

CO_2_ concentration in the red blood cells (rbc) can be calculated analogously to Equation (7) by using the CO_2_ solubility (α_CO2,rbc_) as well as pH (pH_rbc_) and the negative logarithmic equilibrium constant for the overall CO_2_ hydration reaction (pK_rbc_) within the red blood cells:

c_CO2,rbc_ [mmol CO_2_/L] = α_CO2,rbc_ × p_CO2_ × (1 + antilg(pH_rbc_ − pK_rbc_)) = 0.195 [mmol CO_2_/L/kPa] × p_CO2_ [kPa] × (1 + antilg(pH_rbc_ [−] − pK_rbc_) [−]) (9)

The pH in red blood cells is determined by pH of plasma and oxygen saturation of blood (S_O2_):

pH_rbc_ = 7.19 + 0.77 × (pH_pl_ − 7.4) + 0.035 × (1 − S_O2_) = 7.19 [−] + 0.77 [−] × (pH_pl_ [-] − 7.4 [−]) + 0.035 [−] × (1 [−] − S_O2_ [−]) (10)

The negative logarithmic equilibrium constant within the red blood cells is computed based on pH of red blood cells and oxygen saturation of blood:

pK_rbc_ = 6.125 − lg(1 + antilg(pH_rbc_ − 7.84 − 0.06 − S_O2_)) = 6.125 [−] − lg(1 + antilg(pH_rbc_ [−] − 7.84 [−] − 0.06 [−] − S_O2_ [−])) (11)

The total CO_2_ concentration can then be determined using the CO_2_ concentration in plasma (c_CO2,pl_) and red blood cells (c_CO2,rbc_) which are weighted individually with the hematocrit (ϕ):

c_CO2,total_ [mL STP CO_2_/mL] = (c_CO2,pl_ × (1 − ϕ) + c_CO2,rbc_ × ϕ) ÷ 1000 × V_M_ = (c_CO2,pl_ [mmol CO_2_/L] × (1 [−] − ϕ [−]) + c_CO2,rbc_ [mmol CO_2_/L] × ϕ [−]) ÷ 1000 × 22.56 [mL STP CO_2_/mmol CO_2_] (12)

While in the original model the hematocrit is estimated based on hemoglobin concentration (c_tHb_) it could also be measured directly by the BGA. In this study, the measured hematocrit (Hct) was used instead of the estimated hematocrit (ϕ), as direct measurement can be considered more accurate.

ϕ [−] = c_tHb_ ÷ c_tHb,rbc_ ≈ Hct = c_tHb_ [mmol Hb/L] ÷ 21.00 [mmol Hb/L] ≈ Hct [−] (13)

#### 2.4.5. Zierenberg et al.

Similarly to Siggaard-Andersen et al., Zierenberg et al. [23] calculated the CO_2_ content of blood plasma (c_CO2,pl_) and red blood cells (c_CO2,rbc_) separately. The total CO_2_ content in blood plasma is divided into physically dissolved CO_2_ and CO_2_ bound as bicarbonate. The solubility of CO_2_ in plasma (β_pl,CO2_) equals with 3.07 × 10^−5^ mol/mmHg/L the parameter K_s_ (May model, Equation (6)). For calculation of bicarbonate concentration, the equilibrium constant for the overall CO_2_ hydration reaction K_1_ and the pH in blood is used. Hereby, the negative decadic logarithm of K_1_ resembles the negative logarithmic equilibrium constant (pK_pl_, pK_rbc_) of the model proposed by Siggaard-Andersen.

c_CO2,pl_ [mol CO_2_/L] = (1−Hct) × β_pl,CO2_ × p_CO2_ + (1−Hct) × β_pl,CO2_ × K_1_ × p_CO2_ ÷ 10^−pH^ = (1−Hct [−]) × 3.07 × 10^−5^ [mol CO_2_/L/mmHg] × p_CO2_ [mmHg] + (1−Hct [−]) × 3.07 × 10^−5^ [mol CO_2_/mmHg/L] × 7.43 × 10^−7^ [−] × p_CO2_ [mmHg] ÷ 10^−pH [^^−]^ (14)

The total CO_2_ content in red blood cells is divided into physical dissolved CO_2_, CO_2_ bound as bicarbonate, and CO_2_ bound to hemoglobin. The concentration of dissolved CO_2_ and bicarbonate are generally calculated analogously to Equation (14). Only the bicarbonate concentration is multiplied by the Gibbs–Donnan ratio for electrochemical equilibrium across the red blood cell membrane (R_rbc_).

c_CO2,rbc_ [mol CO_2_/L] = Hct × β_rbc__,CO2_ × p_CO2_ + Hct × β_rbc__,CO2_ × R_rbc_ × K_1_ × p_CO2_ ÷ 10^−pH^ + 4 × Hct × Hb_rbc_ × S_HbCO2_ (15)

The last term in Equation (15) represents the amount of CO_2_ bound to hemoglobin. It can be calculated by using the hematocrit (Hct), the hemoglobin concentration in red blood cells (Hb_rbc_), and the CO_2_ saturation of hemoglobin (S_HbCO2_). S_HbCO2_ can be calculated as proposed by Dash et al. [24]. Based on the given model, the fraction of CO_2_ bound to hemoglobin is below 1% of total CO_2_ content. Hence the last term in Equation (15) was neglected in this study, reducing Equations (15) and (16).

c_CO2,rbc_ [mol CO_2_/L] = Hct × β_rbc__,CO2_ × p_CO2_ + Hct × β_rbc__,CO2_ × R_rbc_ × K_1_ × p_CO2_ ÷ 10^−pH^ = Hct [−] × 2.13 × 10^−5^ [mol CO_2_/L/mmHg] × p_CO2_ [mmHg] + Hct [−] × 2.13 × 10^−5^ [mol CO_2_/L/mmHg × 0.69 [−] × 7.43 × 10^−7^ [−] × p_CO2_ [mmHg] ÷ 10^−pH [−]^ (16)

Solubility of CO_2_ in red blood cells (β_rbc,CO2_ − 2.13 × 10^−5^ mol CO_2_/L/mmHg) is comparable to the solubility given by Siggaard-Andersen et al. (α_CO2,rbc_ − 2.60 × 10^−5^ mol CO_2_/L/mmHg). Total CO_2_ concentration can be calculated as the sum of CO_2_ content stored in blood plasma and red blood cells:

c_CO2,total_ [mL STP CO_2_/mL] = (c_CO2,pl_ + c_CO2,rbc_) × V_M_ = (c_CO2,pl_ + c_CO2,rbc_) [mmol CO_2_/mL] × 22.56 [mL STP CO_2_/mmol CO_2_] (17)

### 2.5. Sensitivity Analysis

The influence of the model input parameters, as well as blood flow rate and CO_2_ removal rate, on the prediction performance of the CO_2_ solubility models was examined using the Spearman correlation coefficient (SCC) [25]. Additionally, SCCs were used to analyze the dependency of the sweep flow-based CO_2_ removal rate from blood parameters. SCC values range between −1 and 1, with 0 implying no correlation. A SCC of 1 or −1 implies an exact monotonic relationship. Positive SCC values in Tables 2 and 3 denote that with an increase of the respective parameter the prediction error increases. Negative SCC values in Tables 2 and 3 denote that with an increase of the respective parameter the prediction error decreases. Analogously, SCCs are listed in Table 4 for the dependency of the CO_2_ removal rate on the drop of CO_2_ partial pressure (Δp_CO2_), the drop of bicarbonate (Δc_HCO3_^−^), and the increase of pH (ΔpH).

### 2.6. Statistical Testing

A particular interest of this study was to evaluate whether there is a statistically significant difference in the prediction capabilities of the individual CO_2_ solubility models.

Firstly, the homogeneity of variances between in vitro bovine and in vivo porcine blood data was asserted using Levene’s test [26]. This was done in particular for the prediction error (relative deviation of blood-based from sweep flow-based CO_2_ removal prediction). Based on Levene’s test results, Welch’s *t*-test was chosen to test for significance between the means of two groups, as it is a robust method to compare unequal sample sizes and variances [27].

Welch’s *t*-test was applied for each CO_2_ solubility model to examine the difference between prediction error and inlet pH recorded in in vitro bovine and in vivo porcine blood trials, the difference between sweep gas-based and blood-based CO_2_ removal prediction, and whether adaption of Loeppky model parameters gives a significant improvement in model accuracy.

For May, Siggaard-Anderson, and Zierenberg models, Welch’s *t*-test was utilized to investigate the effect of bicarbonate computation on model performance. Additionally, Levene’s test was used to examine the effect of the bicarbonate computation on the prediction error variance.

To test whether any of the four CO_2_ solubility models perform superiorly, the Games-Howell post-hoc test was applied [28]. For all statistical tests, values of *p* < 0.05 were considered statistically significant.

## 3. Results and Discussion

### 3.1. Accuracy of Sweep Flow-Based CO_2_ Prediction Method

As the suitability of the four different CO_2_ solubility models is evaluated based on the deviation of the blood-based CO_2_ removal prediction from the sweep flow-based CO_2_ removal prediction, the accuracy of the sweep flow-based method is quantified and discussed in the following section. Sweep flow-based CO_2_ removal is determined by measuring two parameters, the sweep flow rate (Q_sweep_) and the CO_2_ concentration of outgoing sweep flow. CO_2_ concentration of ingoing sweep flow was assumed zero as medical O_2_ was used as sweep fluid, Equation (18).

Q_CO2,sweep_ = Q_sweep_ × Δc_CO2,_ with c_CO2,inlet_ = 0 (*medical O_2_*): Q_sweep_ × c_CO2,outlet_ (18)

**Figure 3:**
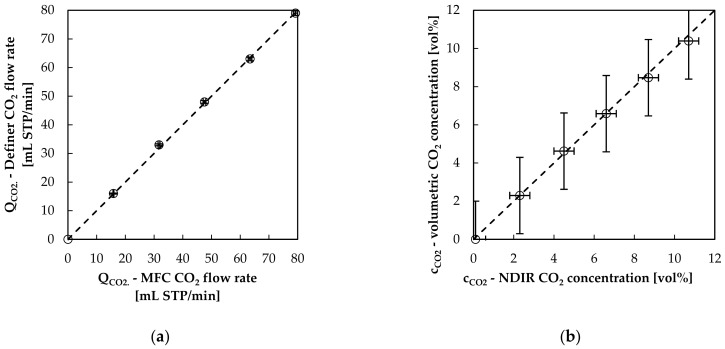
Comparison of (**a**) CO_2_ flow rate determined with volumetric piston stroke meter and mass flow controllers (MFCs) and (**b**) CO_2_ concentration determined volumetrically and with non-dispersive infrared spectroscopy (NDIR).

Sweep flow rate (Q_sweep_) was measured using a high accuracy volumetric piston stroke meter (Defender 510, Bios DryCal). According to the manufacturer, the device has a measurement error of 1% of reading. It is quoted as a calibration method by the Occupational Safety and Health Administration of the United States Department of Labor [29]. Volumetric piston stroke meters do not require a calibration for the gas flow composition [30], which varies during the course of experiments. Accuracy of the piston stroke meter was checked via the mass flow controllers (MFCs) (GF40, Brooks) (Figure 3a). The flow rates determined with the piston stroke meters and the MFCs deviated in average by 1.3%.

CO_2_ concentration of outgoing sweep flow (c_CO2,outlet_) was measured using an NDIR gas analyzer (BINOS 100 M, Emerson). According to the manufacturer the measurement error amounts to 1% of full scale (50 vol%). Before every trial, a two-point calibration at 0 and 5 vol% CO_2_ concentration was conducted to increase the accuracy of the NDIR analyzer. Accuracy of the NDIR analyzer was checked in preliminary studies by measuring the sweep gas flow with CO_2_ and without CO_2_ introduced into sweep flow side of the measurement set up. This was done with the volumetric piston stroke meter. The difference between the two flow rates (with CO_2_ and without CO_2_) was used to calculate the CO_2_ concentration. The volumetrically determined CO_2_ concentration and the CO_2_ concentration measured with NDIR are compared in Figure 3b. The average deviation amounts to 1.7% of volumetrically measured CO_2_ concentration and is insofar below the manufacturer’s specifications.

Utilizing Equation (18) and the measured average errors (sweep flow rate: 1.3%, CO_2_ concentration: 1.7%) the total error of the sweep flow-based method (Q_CO2,sweep,error_) can be estimated to 3% of predicted CO_2_ removal (3% of reading):

Q_CO2,sweep_ + Q_CO2,sweep,error_ = Q_sweep_ × 1.013 × c_CO2,outlet_ × 1.017 = Q_CO2,sweep_ + Q_CO2,sweep_ × 0.03 (19)

In addition to the high accuracy of the measurement devices, the sweep flow-based method has the following principal advantages over the blood-based method:

- The CO_2_ concentration at the sweep flow inlet of the oxygenator can be assumed to be zero, eliminating measurement errors in determining the inlet concentration (necessary for blood-based method).
- NDIR devices are on-line measurement systems (approximate response time of 2 s) while BGAs mostly work off-line (approximate measurement duration 2 min). Consequently, BGA blood samples have to be drawn manually, increasing the risk of errors during sampling.
- No CO_2_ solubility model is necessary in the sweep flow-based method. In this respect, the error introduced by the model and the measurement uncertainties of the additionally required model parameters are not applicable.

Furthermore, gas leakage from the experimental circuit was assessed by examining the total volumetric balance of ingoing and outgoing gas flows. Ingoing flow rates were set by the mass flow controllers, outgoing flow rates were checked with a volumetric piston stroke meter. The volumetric balance of in- and outgoing gases closes within a 1% error margin.

### 3.2. Accuracy of the Blood-Based CO_2_ Prediction Method

To quantify measurement errors on the blood side, the in vitro water tests were assessed. Since the chosen Henry coefficient can be regarded as relatively accurate, the CO_2_ solubility model error should be reasonably small. Due to small errors of the volumetric balance and the Henry model, as well as reasonable measurement accuracy on the sweep gas side (Section 3.1), the deviation of the CO_2_ removal determined for water via the sweep flow-based and blood-based methods can be mostly allocated to measurement errors of the BGA, measurement errors of the ultrasound flowmeter, and errors introduced by the experimental procedure. These errors can be summarized as blood side measurement errors.

**Figure 4:**
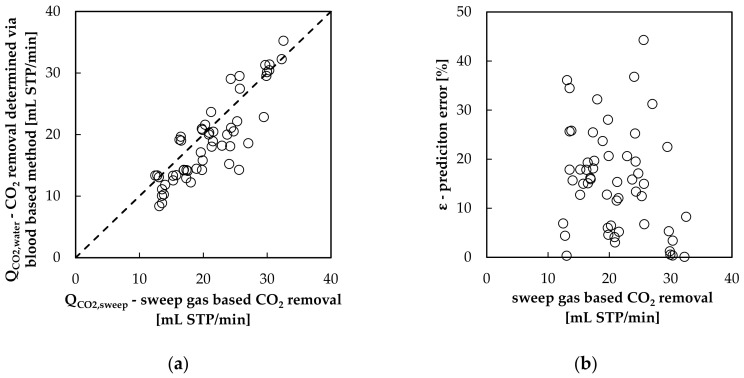
Performance of the Henry law applied for CO_2_ dissolved in water: (**a**) comparison of blood-based to sweep flow-based CO_2_ removal prediction methods and (**b**) prediction error ε in dependency from CO_2_ removal rate.

In Figure 4 the prediction performance of the Henry model, describing the CO_2_ solubility in water, can be examined. 

**Figure 5:**
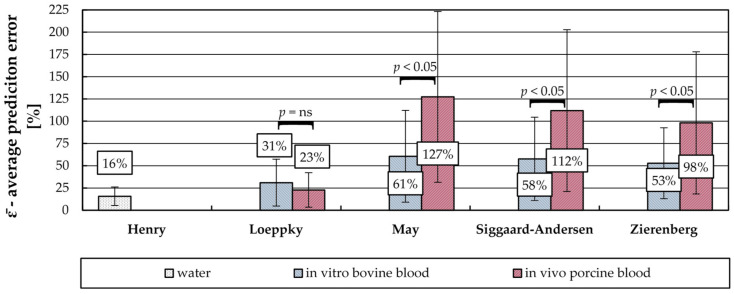
Average prediction error of the different CO_2_ solubility models for in vitro tests with water and bovine blood as well as in vivo tests with porcine blood. The error bars show the standard deviation. Difference of average prediction error between test series was tested for significance with Welch’s *t*-test. Games-Howell test gives that prediction error of Loeppky model is significantly lower (*p*-value < 0.01) than that of the other models for both in vitro and in vivo tests.

Figure 4a shows that the sweep flow-based and blood-based methods match reasonably. In contrast to the findings of Barret et al. [16] the prediction error (ε–Section 2.3) shows no detectable dependency on the amount of CO_2_ removed by the oxygenator (Figure 4b). The average deviation between the sweep flow-based and blood-based method ($\bar{\varepsilon }$) amounts to 16% (Figure 5). This benchmark of 16% can be considered as a reasonable approximation of the blood side measurement error.

CO_2_ solubility models for blood can exceed this benchmark for the average prediction error because of two main reasons. First, due to model errors induced by the respective model itself. Second, due to propagation and possible amplification of BGA measurement errors of additional model input parameters (e.g., c_HCO3_^−^, Hct, pH, S_O2_). The second type of error–propagation of uncertainty–is unavoidably introduced by the mathematical model and is dependent on the used measurement equipment. The prediction error (ε) used in this study to evaluate model suitability includes the model error and the propagation of uncertainty. Consequently, the prediction errors as well as the presented evaluation of the individual solubility models depend on the accuracy of the BGA device used.

However, the influence of the BGA device on the solubility model evaluation can be regarded as small. Roels et al. [31] measured arterial blood samples from 34 dogs using four different BGA devices (Cobas b-123 POC system, IRMA TruPoint, Indexx VetStat and ABL80 FLEX). Only the pH measured by Indexx deviated significantly (*p*-value < 0.01). Nevertheless, the average deviation of pH (Indexx to other BGAs) remains acceptably low and equals 0.019 at a pH of 7.369. The p_CO2_ measured with Cobas and ABL80 deviated significantly (*p*-value < 0.05) from the p_CO2_ measured with IRMA and Indexx. The average deviation of p_CO2_ (Cobas, ABL80 to IRMA, Indexx) is also acceptable and equals 3.1 mmHg at a p_CO2_ of 40.6 mmHg. Based on the reported deviations for pH and p_CO2_ it can be assumed that other BGAs would give qualitatively comparable prediction errors and hence lead to a similar assessment of the solubility models.

### 3.3. Average CO_2_ Removal Prediction Error

The performance of a CO_2_ solubility model can be evaluated by calculating the deviation of the blood-based CO_2_ removal from the sweep flow-based CO_2_ removal (prediction error ε, Section 2.3). In Figure 5 the average deviations ($\bar{\varepsilon }$) of the four presented models (Section 2.4) are compared for in vitro bovine and in vivo porcine blood tests. Additionally, the average deviation for water and the corresponding Henry law is illustrated as a benchmark for a model with desirable low model error (Section 3.2).

For both series of experiments (in vitro bovine and in vivo porcine) and all four examined CO_2_ solubility models, the average prediction error ($\bar{\varepsilon }$) of the blood-based method significantly exceeded the measurement error of the sweep flow-based method (Welch’s *t*-test, *p*-value < 0.01). Of all presented models the simplest model proposed by Loeppky shows the lowest mean deviation between sweep flow-based and blood-based CO_2_ prediction (prediction error). Games-Howell test gives that the average prediction error of the Loeppky model is significantly lower (*p*-value < 0.01) than that of the other models for both in vitro bovine blood and in vivo porcine blood trials.

In the Loeppky model the calculation of the CO_2_ concentration is based only on a single value, the CO_2_ partial pressure. The mean deviation of the Loeppky model equals 31% for in vitro bovine and 23% for in vivo porcine experiments. The mean deviation of the Henry model is hereby exceeded by 15%-points for in vitro bovine and 7%-points for in vivo porcine trials. This deviation between Henry and Loeppky can be considered as a reasonable approximation of the Loeppky model error as both models underly comparable p_CO2_ measurement errors of the BGA. Welch’s *t*-test gives that the Loeppky model does not perform significantly better for in vivo porcine or in vitro bovine blood data (*p*-value = 0.1).

The second simplest model is the May model. In addition to the CO_2_ partial pressure, it uses the bicarbonate concentration, which in this study was taken directly from the BGA measurements. The explicit consideration of the bicarbonate concentration leads to a reduction of the prediction accuracy. The mean deviation of the May model equals 61% for in vitro bovine experiments and 127% for in vivo porcine experiments. The Welch’s *t*-test gives that the May model performs significantly better for in vitro tests with bovine blood than for in vivo tests with porcine blood (*p*-value = 8.6 × 10^−4^).

The more complex models (Siggaard-Anderson, Zierenberg) also explicitly consider the bicarbonate concentration. In contrast to the May model, the bicarbonate concentration is calculated directly using pH (Zierenberg) or pH and S_O2_ (Siggaard-Anderson). Additionally, the distribution of the total CO_2_ content on red blood cells and blood plasma is mathematically considered using the hematocrit. However, Siggaard-Anderson and Zierenberg models perform similarly to the May model. No significant difference in prediction error was detected when comparing these models (Games-Howell test, *p*-value > 0.05). The mean deviation of Siggaard-Anderson model equals 58% for in vitro bovine and 112% for in vivo porcine blood data. The mean deviation of Zierenberg model equals 53% for in vitro bovine and 98% for in vivo porcine blood data. Based on the available data, consideration of the CO_2_ content distribution on red blood cells and blood plasma does not substantially improve prediction performance. Similar to the May model, the Welch’s *t*-test gives that Siggaard-Anderson (*p*-value = 3.5 × 10^−3^) and Zierenberg model (*p*-value = 5.1 × 10^−3^) perform significantly better for the in vitro bovine than for the in vivo porcine blood experiments.

In general, the average deviation of blood-based CO_2_ removal prediction from the sweep flow-based CO_2_ removal prediction is high. One reason for this could be that the CO_2_ solubility models were developed–or at least use solubility parameters–for human blood. Other than the animal species chosen for the in vitro and in vivo trials, the publications of Loeppky [20], May [11], Siggaard-Anderson [22], and Zierenberg models [23] give no indication that the solubility models were applied outside their validity limits. However, May’s model was proposed to determine total CO_2_ removal during respiratory dialysis. With this separation technique, CO_2_ removal is based on separation of bicarbonate by a hemodialysis membrane [32]. Consequently, when compared to membrane oxygenation, a larger decrease in bicarbonate concentration can be expected (Section 2.4). Nevertheless, our data suggest that accurate determination of the CO_2_ removal performance of an oxygenator can only be guaranteed with the sweep flow-based CO_2_ removal prediction method. Even with a suitable solubility model, an average error of approximately 30% remains. This is in agreement with findings of Barret et al. [16], who observed a deviation of 20% at similar ratios between CO_2_ removal rate and blood flow rate (Section 1). The Loeppky model, when chosen for in vivo porcine blood experiments fits well within this range.

**Figure 6:**
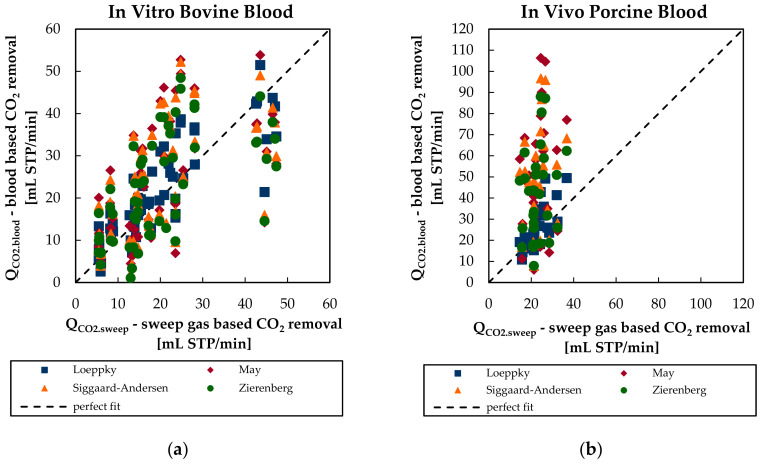
Blood-based CO_2_ removal rate of the four CO_2_ solubility models in dependency of the sweep flow-based CO_2_ removal rate for (**a**) in vitro bovine blood tests and (**b**) in vivo porcine blood tests.

The tendency of a model to under- or overpredict the CO_2_ removal rate can be assessed when plotting the blood-based CO_2_ removal rate over the sweep flow-based CO_2_ removal rate (Figure 6). For in vitro bovine blood tests (Figure 6a), the Loeppky model tends to slightly overpredict the CO_2_ removal rate. In contrast, all other models evaluated (May, Siggaard-Anderson, and Zierenberg) show both over- and underprediction of the CO_2_ removal rate.

For in vivo porcine blood tests (Figure 6b), the Loeppky model also yields a slight overprediction of the CO_2_ removal rate. May, Siggaard-Anderson, and Zierenberg models largely overpredict the CO_2_ removal when applied to the porcine blood data. The increased scattering of these three models for in vivo porcine blood experiments is discussed in Section 3.4.

### 3.4. Variation of CO_2_ Removal Prediction Error

**Figure 7:**
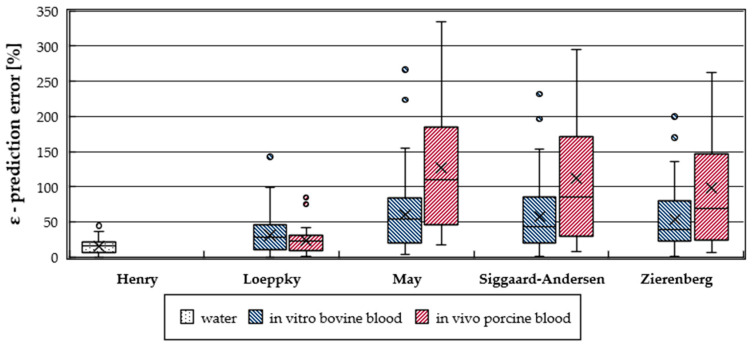
Prediction error distribution of all measurements compared for the different CO_2_ solubility models and experimental trials–in vitro bovine and in vivo porcine.

While the average prediction error ($\bar{\varepsilon }$) provides information about the overall model accuracy, the variation of the prediction error allows examination of the stability and reliability of a model. To illustrate the variation of the available data, prediction errors of the different CO_2_ solubility models are visualized by a box plot (Figure 7) for both in vitro bovine and in vivo porcine trials.

Levene’s test gives that prediction error variation is significantly increased for porcine blood in vivo trials for May (*p*-value = 1.7 × 10^−4^), Siggaard-Anderson (*p*-value = 1.5 × 10^−4^), and Zierenberg (*p*-value = 2.3 × 10^−4^) models. No significant difference in prediction error variation was recorded for the Loeppky model (Levene’s *t*-test, *p*-value = 1.8 × 10^−1^). For all models the relative standard deviations of the prediction errors (ε) of in vitro and in vivo trials are comparable (Table 1).

The high absolute variation of the prediction error of the May, Siggaard-Anderson, and Zierenberg models can be attributed to the explicit calculation of the bicarbonate concentration. May, Siggaard-Anderson, and Zierenberg models as well as the BGA calculate the bicarbonate concentration based on the Henderson-Hasselbalch equation [21], which allows the calculation of bicarbonate concentration (c_HCO3_^−^) based on concentration of dissolved CO_2_ (c_CO2_) and pH [33]. In the Henderson-Hasselbalch equation (Equation (20)) pK represents the negative logarithmic equilibrium constant for the overall CO_2_ hydration reaction and α_CO2_ the CO_2_ solubility of blood:

c_HCO3_^−^ = c_CO2_ × antilg(pH − pK) = α_CO2_ × p_CO2_ × antilg(pH − pK) (20)

**Figure 8:**
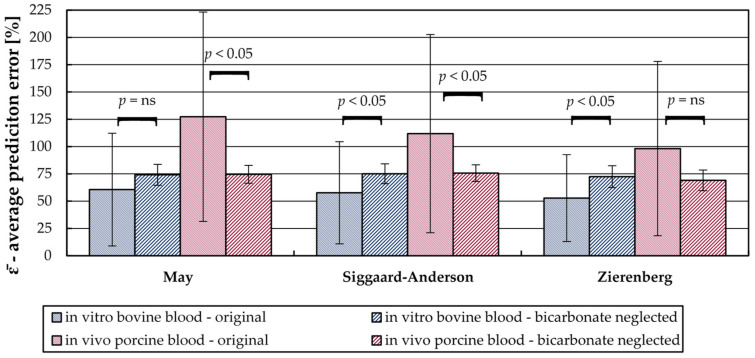
Average prediction error of the different solubility models when including (original) and neglecting the calculation of the bicarbonate content in blood. The error bars show the standard deviation. Difference of average prediction error between original model and model with neglected bicarbonate calculation was tested for significance with Welch’s *t*-test.

Neglecting the bicarbonate term in the model of May, Siggaard-Anderson, and Zierenberg significantly reduces the variation in prediction error (Levene’s test, *p*-value < 0.01) for both in vitro bovine and in vivo porcine experiments (Figure 8). For the in vitro bovine blood data, the variation of the three models reduces to 20% of the variations of the original models. For in vivo porcine blood data, the variations reduce to approximately 15% of the variations of the original models.

When neglecting the bicarbonate term, the average prediction error of the three models is comparable. They range from 72 to 74% and from 69 to 76% for in vitro bovine and in vivo porcine blood data, respectively. These small deviations between the models can be explained by the solubility coefficients for physical dissolved CO_2_. As described in Section 2.4, all three models use similar values.

Based on the in vivo porcine blood data, the prediction error can be reduced significantly for May (Welch’s *t*-test, *p*-value = 4.6 × 10^−3^) and Siggaard-Anderson (Welch’s *t*-test, *p*-value = 3.4 × 10^−2^) models by neglecting the bicarbonate term. The average prediction error ($\bar{\varepsilon }$) reduces from 100% (Zierenberg) and 130% (May) to a value of approximately 75%. No significant reduction was recorded for the Zierenberg model (Welch’s *t*-test, *p*-value = 5.3 × 10^−2^).

For bovine blood experiments the average prediction error ($\bar{\varepsilon }$) increases from 61% (May), 58% (Siggaard-Anderson), and 53% (Zierenberg)–to a value of approximately 73%. This increase is significant for Siggaard-Anderson (Welch’s *t*-test, *p*-value = 7.4 × 10^−3^) and Zierenberg (Welch’s *t*-test, *p*-value = 5.6 × 10^−4^). No significant increase was recorded for the May model (Welch’s *t*-test, *p*-value = 5.5 × 10^−2^).

**Figure 9:**
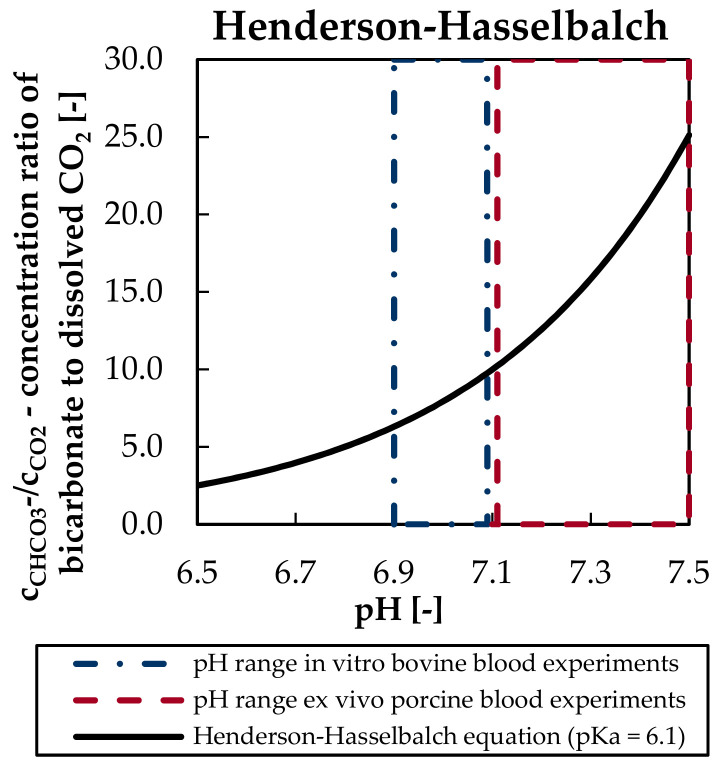
Ratio of bicarbonate to dissolved CO_2_ concentration in dependency of the pH.

Increased prediction errors and prediction error variance of the in vivo porcine blood experiments could be partially caused by the use of the Henderson-Hasselbalch equation. According to the equation, the ratio of bicarbonate concentration and CO_2_ partial pressure is exponentially dependent on pH (Figure 9). Consequently, measurement errors of the CO_2_ partial pressure and the pH are successively amplified with increasing pH.

This could explain the increased prediction error and prediction error variance of the in vivo studies with porcine blood as they showed pH values on a higher level and wider range (7.1–7.5) than the in vitro trials with bovine blood (6.9–7.1). Welch’s *t*-test confirms that mean inlet pH of in vivo porcine and in vitro bovine trials deviates significantly (*p*-value < 0.01). The publications of May [11], Siggaard-Anderson [22], and Zierenberg [23] models give no indication that the correlations for determination of bicarbonate concentration were applied outside their validity limits.

Additionally, less controllable conditions of in vivo tests could contribute to the higher prediction error and prediction error variance.

### 3.5. Sensitivity Study

The influence of input parameters on the prediction performance was quantified by computing the Spearman correlation coefficient (SCC). The SCC values between the model prediction error and different blood parameters are summarized in Table 2 (in vitro bovine blood) and Table 3 (in vivo porcine blood). Additionally, the SCCs between the prediction error and the CO_2_ removal rate as well as the prediction error and the blood flow rate are given. For in vitro bovine blood data, the prediction error shows no distinct dependency on the given parameters. The SCC values range between 0.23 for the prediction performance of Loeppky and the hematocrit, to −0.31 for the prediction performance of Loeppky and the CO_2_ removal rate. SCC values of model input parameters are not particularly elevated or lowered compared to SCC values of non-input parameters.

Although the pH influences the sensitivity of the Henderson-Hasselbalch equation for calculation of the bicarbonate concentration (Section 3.4), the SCC of pH and the prediction error are low for the May, Siggaard-Anderson, and Zierenberg models. It ranges from 0.05 to 0.06 (Table 3). The prediction error of the Loeppky model decreases with increasing pH (SCC = −0.31). However, it should be noted that the Loeppky model does not use pH as input parameter. In contrast to Barret et al. [16], no significant influence of the CO_2_ removal rate on the prediction performance can be determined for bovine and porcine blood trials. This may be partly due to the limited range of CO_2_ removal rates measured in this study.

Compared to in vitro trials with bovine blood, SCC values determined in vivo with porcine blood show a relatively strong dependency of the model prediction performance on the hematocrit (0.60–0.63). This comprises models including (Siggaard-Anderson and Zierenberg) and excluding (May) the hematocrit as an input parameter. The prediction error of May, Siggaard-Anderson, and Zierenberg increases with the hematocrit. The Loeppky model, which similar to May does not include the hematocrit as an input parameter, shows a low dependency of the prediction performance on the hematocrit (SCC = 0.14).

Table 4 shows the SCC for the CO_2_ removal rate and selected process parameters for in vitro bovine and in vivo porcine blood data (Section 2.5). Here, Δ denotes the change of the corresponding parameter from blood inlet to blood outlet of the membrane module. The SCC were calculated for all measurement points with a blood flow rate of approximately 1000 mL/min (980–1100 mL/min).

**Figure 10:**
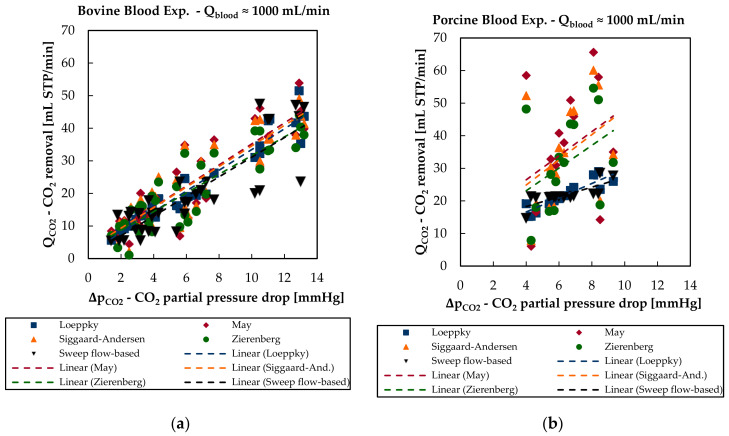
Sweep flow-based CO_2_ removal and blood-based CO_2_ removal rate of the four different CO_2_ solubility models in dependency of the CO_2_ partial pressure drop over the membrane module: (**a**) in vitro bovine blood trials and (**b**) in vivo porcine blood trials.

As can be expected, there is a strong dependency between the CO_2_ removal rate and the drop of CO_2_ partial pressure over the membrane module for both experimental campaigns. The dependency is more pronounced for in vitro bovine blood (SCC = 0.90) than for in vivo porcine blood data (SCC = 0.75). For in vitro bovine blood experiments, the dependence of the CO_2_ removal rate on the CO_2_ partial pressure drop can be qualitatively described by all four solubility models (Figure 10a). For in vivo porcine blood data, only the Loeppky model is capable of reproducing this dependency (Figure 10b).

**Figure 11:**
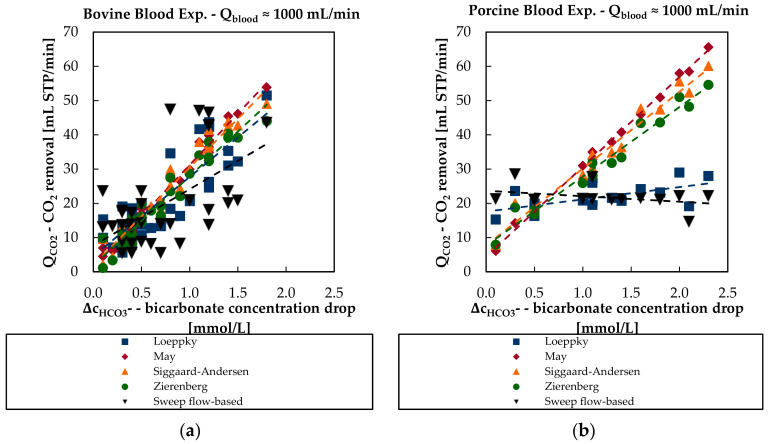
Sweep flow-based CO_2_ removal and blood-based CO_2_ removal rate of the four different CO_2_ solubility models in dependency of the bicarbonate concentration drop over the membrane module: (**a**) in vitro bovine blood trials and (**b**) in vivo porcine blood trials.

The dependency of the CO_2_ removal rate from the drop of the bicarbonate concentration is less distinctive. SCC of the in vitro bovine blood trials equals 0.57 and SCC of in vivo porcine blood trials equals −0.02. For in vitro bovine blood data, the increase of the CO_2_ removal rate with a higher bicarbonate concentration drop can be qualitatively described by the solubility models (Figure 11a). 

The negative SCC for in vivo porcine blood data is physically not sound and could be attributed to scattering of the data. Additionally, the slope between the CO_2_ removal rate and the drop of the bicarbonate concentration is low, producing a small SCC value. In contrast, May, Siggaard-Anderson, and Zierenberg models predict a stronger dependency of CO_2_ removal rate on the drop of the bicarbonate concentration (Figure 11b).

The SCC values of the obtained data suggest that the CO_2_ removal rate is more sensitive to the change of CO_2_ partial pressure than to the change of bicarbonate concentration. As computation of bicarbonate concentration introduces additional uncertainty in the prediction accuracy (Section 3.4), our data indicate that CO_2_ partial pressure is more suitable than bicarbonate for accurate prediction of the CO_2_ removal rate. Additionally, the SCC values also indicate that the CO_2_ removed by the prototype oxygenator was to a large extent physically dissolved.

**Figure 12:**
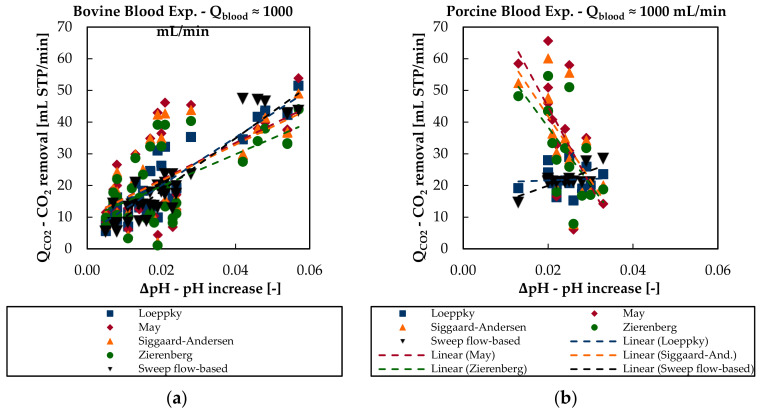
Sweep flow-based CO_2_ removal and blood-based CO_2_ removal rate of the four different CO_2_ solubility models in dependency of the pH increase over the membrane module: (**a**) in vitro bovine blood trials and (**b**) in vivo porcine blood trials.

There is a stronger correlation between CO_2_ removal rate and pH increase (ΔpH–Table 4) for in vitro bovine than for in vivo porcine blood data. SCC of in vitro bovine blood tests equals 0.87 and SCC of in vivo porcine blood tests equals 0.37. The Loeppky model is capable of quantitively reproducing the CO_2_ removal rate for different levels of pH increase in both trials (Figure 12). For in vitro bovine blood data, May, Siggaard-Anderson, and Zierenberg models allow a rough qualitative description of the CO_2_ removal rate dependency on the pH increase. However, they erroneously predict a decrease of the CO_2_ removal with higher ΔpH for in vivo porcine blood experiments.

### 3.6. Adaption of Loeppky Model Parameters

**Figure 13:**
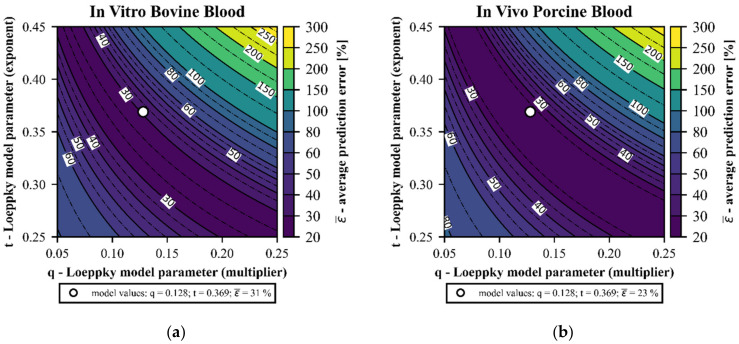
Average prediction error ($\bar{\varepsilon }$) of the Loeppky model as a function of the two empirical model parameters q and t: (**a**) in vitro bovine blood trials and (**b**) in vivo porcine blood trials.

Dependency of Loeppky model performance on the two empirical model parameters q and t was investigated. Figure 13 shows the average prediction error ($\bar{\varepsilon }$) of the Loeppky model as a function of q ant t for the in vitro bovine and in vivo porcine blood data. As can be seen in the contour plots, the original parameters already give an average prediction error close to the minimum value. The average prediction error determined for the in vitro bovine blood trials can be reduced from 31% (○–Figure 13a) to 24%. Analogously, average prediction error for the in vivo porcine blood trials can be reduced from 23% (○—Figure 13b) to 21%.

However, Welch’s *t*-test gives that minimum average prediction errors of in vitro bovine and in vivo porcine blood data do not deviate statistically significantly (*p*-value > 0.05) from the average prediction errors of the original model parameters. Consequently, the original parameters can be considered as generic and suitable for bovine and porcine blood.

## 4. Conclusions

In this study, we investigated performances of four different CO_2_ solubility models for bovine blood in in vitro and porcine blood in in vivo studies. To examine the respective model performance, the CO_2_ removal rate was determined using two methods. First, based on the increase of CO_2_ concentration in sweep flow and, second, based on the decrease of CO_2_ concentration in blood. While the first method (sweep flow-based) can be considered sufficiently accurate (measurement error approx. 3% of reading), the second method (blood-based) depends on a suitable CO_2_ solubility model in addition to BGA measurements. In this work, the errors introduced by the CO_2_ solubility models were quantified by computing the deviation of the blood-based CO_2_ removal rate from the sweep flow-based CO_2_ removal rate (prediction error).

Statistical analyzes of the results show that the simplest CO_2_ solubility model (Loeppky) is in general superior and more robust as compared to three different models with added complexity (May, Siggaard-Anderson, and Zierenberg). Additionally, our data suggest that the models proposed by May, Siggaard-Anderson, and Zierenberg perform significantly better for in vitro bovine blood data than for in vivo porcine blood data. Furthermore, they show significantly increased variance of CO_2_ removal prediction error due to computation of bicarbonate concentration via Henderson-Hasselbalch equation.

The best performing model (Loeppky) showed an average deviation of the blood-based CO_2_ removal rate from the sweep flow-based CO_2_ removal rate (average prediction error) of 31% for in vitro bovine blood and of 23% for in vivo porcine blood trials. In contrast to the other models, the difference in model performance between the in vitro bovine and in vivo porcine blood experiments was not significant. Adaptation of the empirical Loeppky model parameters to individual animal species and test procedures allows for no significant improvement of the prediction accuracy. Hence, the original parameter set can be considered as reasonably accurate.

The prediction error of the blood-based method significantly exceeds the measurement error of the sweep flow-based method, regardless of the CO_2_ solubility model chosen. Although the recorded magnitude of the deviation between blood-based and sweep flow-based CO_2_ removal is high, it is consistent with results reported in the literature. A prediction error of up to 30% should be assumed for blood-based CO_2_ removal rate determination, even assuming application of a suitable solubility model. Consequently, for accurate determination of the CO_2_ removal rate of an oxygenator, it is recommended to measure the CO_2_ content in the exhaust gas.

## Figures and Tables

**Table 1:** Relative standard deviation of the prediction error (ε) of the four CO_2_ solubility models in in vitro bovine and in vivo porcine blood trials.

Trial	Loeppky ^1^	May	Siggaard-Andersen	Zierenberg
In vitro bovine blood	0.85	0.85	0.81	0.75
In vivo porcine blood	0.85	0.75	0.81	0.81

^1^ No significant difference in prediction error variation between the two trials (Levene’s Test, *p*-value > 0.05).

**Table 2:** Spearman correlation coefficients between average prediction error ($\bar{\varepsilon }$) and selected parameters calculated for in vitro bovine blood trials and the four CO_2_ solubility models.

Solubility Model	Q_Blood_	Q_CO2_	p_CO2_	c_HCO3_^−^	Hct	pH	S_O2_
Loeppky	0.03	−0.31	−0.02 ^1^	0.04	0.23	0.01	−0.03
May	−0.01	−0.19	0.15 ^1^	0.20 ^1^	0.13	−0.08	0.02
Siggaard-Andersen	−0.05	−0.27	0.05 ^1^	0.14	0.10 ^1^	0.00 ^1^	0.03 ^1^
Zierenberg	−0.07	−0.20	0.10 ^1^	0.13	0.07^1^	−0.04 ^1^	−0.06

^1^ Parameter is an input parameter of the corresponding solubility model.

**Table 3:** Spearman correlation coefficients between average prediction error ($\bar{\varepsilon }$) and selected parameters calculated for in vivo porcine blood trials and the four CO_2_ solubility models.

Solubility Model	Q_Blood_	Q_CO2_	p_CO2_	c_HCO3_^−^	Hct	pH	S_O2_
Loeppky	0.48	0.35	0.32 ^1^	−0.02	0.14	−0.31	−0.05
May	0.50	0.02	0.01 ^1^	0.07 ^1^	0.63	0.05	0.00
Siggaard-Andersen	0.45	0.01	0.00 ^1^	0.09	0.60 ^1^	0.05 ^1^	0.03 ^1^
Zierenberg	0.47	−0.01	−0.02 ^1^	0.06	0.61 ^1^	0.06 ^1^	0.05

^1^ Parameter is an input parameter of the corresponding solubility model.

**Table 4:** Spearman correlation coefficients between CO_2_ removal rate and selected process parameters calculated for in vitro bovine and in vivo porcine blood trials.

Experimental Campaign	Δp_CO2_	Δc_HCO3_^−^	ΔpH
In vitro bovine blood	0.90	0.57	0.87
In vivo porcine blood	0.75	−0.02	0.37

## Data Availability

The data that support the findings of this study are available from the corresponding author upon reasonable request.

## Nomenclature

**Acronyms**	
ARDS	Acute respiratory distress syndrome
BGA	Blood gas analyzer
CFD	Computational fluid dynamics
CO_2_	Carbon dioxide
ECMO	Extracorporeal membrane oxygeantor
NDIR	Non-dispersive infrared photometer
HCO3^−^	Bicarbonate
LPV	Lung protective ventilation
N_2_	Nitrogen
O_2_	Oxygen
PBS	Phosphate buffered solution
SCC	Spearman correlation coefficient
PMP	Polymethylpentene
STP	Standard temperature and pressure (273.15 K, 1 bar)
RBC	Red Blood Cells
**Latin Symbols**	
c_CO2_	Concentration of dissolved CO_2_
c_CO2,pl_	CO_2_ concentration in blood plasma
c_CO2,rbc_	CO_2_ concentration in red blood cells
c_CO2,total_	Total CO_2_ content of blood
c_HCO3_^−^	Bicarbonate concentration
ct_Hb_	Hemoglobin concentration in whole blood
c_tHb,rbc_	Hemoglobin concentration in red blood cells (Siggaard-Anderson model)
Hb_rbc_	Hemoglobin concentration in red blood cells (Zierenberg model)
Hct	Measured hematocrit
K_1_	Equilibrium constant for the overall CO2 hydration reaction
k_H_	Henry coefficient for CO_2_ in water
K_s_	Henry coefficient for the CO_2_ solubility in blood (May Model)
M_CO2_	Molar mass of CO_2_
p_CO2_	CO_2_ partial pressure
pH_pl_	pH in plasma
pH_rbc_	pH in red blood cells
pK	Negative logarithmic equilibrium constant for the overall CO_2_ hydration reaction
pK_pl_	Negative logarithmic equilibrium constant for the overall CO_2_ hydration reaction in blood plasma
pK_rbc_	Negative logarithmic equilibrium constant for the overall CO_2_ hydration reaction within RBC
Q	Empirical parameter of the Loeppky model
Q_blood_	Blood flow rate
Q_CO2,blood_	CO_2_ removal determined with the blood-based prediction method
Q_CO2,sweep_	CO_2_ removal determined with the sweep flow-based prediction method
Q_CO2,sweep,error_	Error of the sweep flow-based CO_2_ removal prediction
q	Empirical parameter of the Loeppky model (multiplier)
Q_sweep_	Sweep flow rate
R_rbc_	Donnan ratio for electrochemical equilibrium across the red blood cell membrane
S_HbCO2_	CO_2_ saturation of hemoglobin
S_O2_	Oxygen saturation
T	Temperature
t	Empirical parameter of the Loeppky model (exponent)
V_M_	Molar volume of CO_2_ at standard temperature and pressure
x, y	Arbitrary variable
**Greek Symbols**	
α_CO2_	Solubility coefficient of CO_2_ in blood
α_CO2,pl_	Solubility coefficient of CO_2_ in blood plasma (Siggaard-Anderson model)
α_CO2,rbc_	Solubility coefficient of CO_2_ in red blood cells (Siggaard-Anderson model)
β_CO2,pl_	Solubility coefficient of CO_2_ in blood plasma (Zierenberg model)
β_CO2,rbc_	Solubility coefficient of CO_2_ in red blood cells (Zierenberg model)
Δc_CO2,blood_	CO_2_ concentration difference between inlet and outlet on the blood side
Δc_CO2,sweep_	CO_2_ concentration difference between inlet and outlet on the sweep fluid side
Δp_CO2_	Drop of CO_2_ partial pressure over the membrane module
Δc_HCO3_^−^	Drop of bicarbonate concentration over the membrane module
ΔpH	Increase of pH over the membrane module
ε	Relative deviation of blood-based from sweep flow-based CO_2_ removal prediction
$\bar{\varepsilon }$	Average relative deviation of blood-based from sweep flow-based CO_2_ removal prediction
ρ_CO2_	Density of CO_2_ at standard temperature and pressure
ρ_water_	Density of water at 310.15 K
ϕ	Estimated hematocrit
